# Probiotics - a helpful additional therapy for bacterial vaginosis

**Published:** 2013-12-25

**Authors:** O Bodean, O Munteanu, C Cirstoiu, D Secara, M Cirstoiu

**Affiliations:** * Department of Obstetrics and Gynecology, Bucharest University Emergency Hospital; ** Department of Orthopedics and Traumatology, Bucharest University Emergency Hospital

**Keywords:** bacterial vaginosis, probiotics

## Abstract

Abstract

Background: Bacterial vaginosis is a condition of unknown etiology, associated with an imbalance of the normal vaginal microbiota, characterized by a high recurrence rate despite of classical therapy solutions. Probiotics are microorganisms, which taken in adequate amounts, are proven to bring health benefits in human and animal bodies, by re-establishing the normal flora at different levels.

Objective: The present article studies the possibility of using probiotic treatment as an adjuvant therapy for nonspecific vaginosis and reducing its recurrence rate.

Methods: We have evaluated the evolution of patients with bacterial vaginosis who received the classical antibiotic therapy and a probiotic product. The study group consisted of 173 non-pregnant, sexually active patients, 20-45 years old, with no additional health problems and no contraceptive undergoing treatment, which have been admitted to the department of Obstetrics and Gynecology of the Bucharest Emergency University Hospital between 1.01.2012-31.12.2012.The bacteriological evaluation was made on cervical and vaginal cultures.

Results: From a total of 173 patients, those who used probiotics oral capsules while taking an antibiotic had lower recurrence rates. More than a half of women who did not use any probiotic product had 3 or more relapse episodes per year. Vaginal capsules with probiotics have also proven to be useful in lowering the recurrence rate, but research is still needed.

Conclusion: Probiotic products are proven to be a helpful adjuvant therapy for bacterial vaginosis, with no adverse outcomes.

## Introduction

The normal bacterial microflora is a complex part of the immune defense system. The interactions between the species which colonize the intestine, the urogenital tract and the health status of the whole body are subjected to intense research. Probiotics are defined as: “living microorganisms, which ingested in adequate amounts, can have beneficial effects in the human body” (FAO 2001). The most frequently studied probiotics are Bifidobacterium and Lactobacillus [**1**]. Probiotics can be used as pills, oral solution drinks, oral and vaginal capsules and can be found in different food products (e.g. yogurt) [**2**]. The most common diseases in which probiotics have been previously used are: diarrhea, iritative bowel syndrome, diarrhea associated with long term antibiotic treatment, infection with Clostridium difficile, bacterial vaginosis [**3**]. 

In healthy individuals, lactobacilli are normally found in the gastrointestinal and urogenital tracts. These bacteria produce peroxide and have a role in proteolysis [**4**]. Lactobacilli produce anti-microbial substances including acidolin, acidophilin, lactocidin and bacteriocin [**5**]. 

Bacterial vaginosis is a disease characterized by a decline in the concentration of lactobacilli and an overgrowth of other microorganisms, especially anaerobic and gram-negative species. The most frequently detected species are: Gardnerella vaginalis, Prevotella species, Porphyromonas species, Bacteroides species, Peptostreptococcus species, Mycoplasma hominis, Ureaplasma urealyticum, Mobiluncus species, Fusobacterium species and Atopobium vaginae. 

The etiology and pathophysiology of the disease are not completely understood and therefore, the classical therapy is not always fully efficient. The bacterial vaginosis is characterized by a high rate of recurrence in sexual active women, the patients having 3 or more relapses each year. In more than half cases, the patients are asymptomatic. The most typical symptom is a foul-smelling profuse vaginal discharge, which intensifies after sexual intercourse [**6**]. 

Women who have bacterial vaginosis seem to have a high concentration of different species of bacteria in their vagina, which can reach the upper genital tract. According to research evidence described by literature, these women have a higher risk in developing pelvic inflammatory disease. 

## Materials and methods

We have evaluated the evolution of patients with bacterial vaginosis who received the classical antibiotic therapy and a probiotic product. The study group consisted of 173 non-pregnant, sexually active patients, 20-45 years old, with no additional health problems and no contraceptive undergoing treatment, which have been admitted to the 3rd department of Obstetrics and Gynecology (O.G.3) of the Bucharest Emergency University Hospital between 1.01.2012-31.12.2012. The patients were divided in 3 groups A, B and C, of which, group A was considered control group and who did not receive any probiotic treatment. All groups received the classical oral therapy with metronidazole 500 mg twice daily for 7 days and topical metronidazole cream for 5 days. Group B received a probiotic product for vaginal use and group C received a probiotic product for oral use. The bacteriological evaluation has been performed on cervical and vaginal cultures taken before treatment, at 2 months and at 3 months after therapy.

The probiotic products we used have been of two types:

- Oral tablets containing Lactobacillus acidophilus 750x106 living organisms, equivalent of 5 mg and Lactobacillus bifidus 250x106 living organisms, equivalent of 3 mg.

- Vaginal capsules containing 10 milliard living cells of a mixture of L. Rhamnosus B, L. Acidophilus, S. Thermophilus and L. Bulgaricus.

The organisms that have been identified in cervical and vaginal cultures of patients before starting therapy, were, ordered by frequency: E. Coli, Klebsiella pneumoniae, Klebsiella oxytoca, Streptococ grup B, Enterobacter spp., Proteus mirabilis, Stafilococcus aureus MRSA.

The antibiotic treatment of bacterial vaginosis consisted of metronidazole 500mg twice daily for 7 days associated with topical metronidazole cream for 5 days.

The patients in group C received the probiotic treatment for 10 days, taking 2 capsules daily at 2 hours after the ingestion of the antibiotic. The patients in group B used 1 vaginal capsule (ovule) daily, for 6 days. The therapy was repeated for 3 consecutive menstrual periods.

## Results

**Fig. 1:**
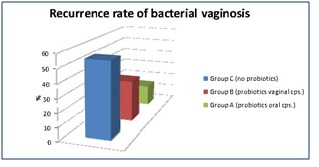
Recurrence rate of bacterial vaginosis

From a total number of 173 patients, those in group A, who did not receive any additional therapy, had a rate of disease recurrence of more than 50% during 12 months (Fig. 1).

In group C, who was administered oral tablets of a probiotic product, the recurrence rate of the disease was of only 15 % in the first 3 months after therapy.

The women who used the probiotic vaginal capsules had a recurrence rate of 30%, but also a low follow-up rate, due to route of administration, which these women have found to be uncomfortable.

The best results of probiotic therapy were noticed in women with E. Coli, Proteus and Klebsiella.

Using an oral probiotic product has lowered the risk of Clostridium infection, as is the case of group C. In group A, who did not receive probiotics, 5 cases of Clostridium infection have been reported.

According to literature, the protective mechanism of probiotics upon normal intestinal and vaginal flora consists of: control of excessive growth of potentially pathogenic bacteria, by modifying pH, the stimulation of the immune system and antioxidant production, stimulation of interleukin-10 production and inhibiting the growth of Clostridium and Candida albicans.

If left untreated, bacterial vaginosis may cause pelvic inflammatory disease, infections after gynecological surgery (hysteroscopy), endometritis and chorioamnionitis (40% of preterm births). There are studies which seem to find a connection between vaginal bacteriosis and a higher risk of acquiring HIV, especially in multiparus women [**7**].

## Conclusion

Bacterial vaginosis is a disease characterized by a disturbance in the normal microbiota of the vagina, leading to a decreased number of lactobacilli and a high recurrence rate due to the incapacity of regenerating and maintaining adequate amounts of lactobacilli after antibiotic treatment.

Antibiotic treatment of bacterial vaginosis is constant, using almost the same therapy schemes for years (metronidazol, tinidazol and clindamycin), but more than half patients have 2-3 relapses per year.

Probiotics seem to be a helpful additional therapy for treating bacterial vaginosis and reestablishing the fragile equilibrium in the intestinal and vaginal microflora, with no adverse effects.

The most effective route of administration of the probiotic, in our case, seems to be oral, even if vaginal capsules have proven to be useful for some patients. More research is needed in this area. We conclude that associating probiotics to the therapy of bacterial vaginosis may be very useful in decreasing the number of recurrence of the disease.

**Disclosure**: None of the authors have a conflict of interest.

**Acknowledgement**:This study was supported by the international project “Development of the translational research infrastructure in molecular and imagistic pathology - MOLIMAGEX”. Project manager: Prof. Catalin Cirstoiu, MD. Responsible for the Obstetrics-Gynecology Department: Assoc. Prof. Monica Cirstoiu, MD
